# Electron Microscopic Radioautographic Study on Protein Synthesis in Hepatocyte Mitochondria of Aging Mice

**DOI:** 10.1100/tsw.2006.265

**Published:** 2006-12-15

**Authors:** Tetsuji Nagata

**Affiliations:** Department of Anatomy and Cell Biology, Shinshu University School of Medicine, Matsumoto 390-8621 and Department of Anatomy, Shinshu Institute of Alternative Medicine, Nagano 380-0816, Japan

**Keywords:** mitochondria, EM radioautography, protein syntheses, mouse hepatocytes, aging

## Abstract

For the purpose of studying the aging changes of intramitochondrial protein synthesis in mouse hepatocytes, 10 groups of aging mice, each consisting of 3 individuals (total 30), from fetal day 19 to postnatal month 24, were injected during development with 3H-leucine, a protein precursor, sacrificed 1 h later, and the liver tissues processed for electron microscopic (EM) radioautography. On EM radioautograms obtained from each animal, the number of mitochondria, the number of labeled mitochondria, and the mitochondrial labeling index labeled with silver grains due to H-leucine showing protein synthesis in each mononucleate hepatocytes were counted and the averages in respective aging groups were compared. From the results, it was demonstrated that the numbers of mitochondria, the numbers of labeled mitochondria, and the labeling indices of intramitochondrial protein syntheses in mononucleate hepatocytes of mice at various ages from embryonic day 19 to postnatal month 24 increased and decreased due to development and aging of animals.

### INTRODUCTION

Macromolecular synthesis, such as nucleic acids, proteins, glucides, and lipids syntheses in nuclei and cell bodies of various kinds of cells in various organs of experimental animals, has been extensively studied for many years using both biochemical and morphological approaches[1,2,3,4]. Among these studies, intramitochondrial nucleic acid syntheses, both DNA and RNA, in mammalian and avian cells were first demonstrated morphologically by the present author and associates by means of electron microscopic (EM) radioautography, with accurate localization in primary cultured cells of the livers and kidneys of mice and chickens *in vitro*[5,6], and then in some other established cell lines, such as HeLa cells[7,8,9,10,11,12] or mitochondrial fractions prepared from *in vivo* cells[13,14]. These phenomena were later commonly found in various cells and tissues not only *in vitro* obtained from various organs *in vivo*[15,16,17,18,19,20,21,22,23], but also *in vivo* cells of various organs such as the salivary gland[24], the liver[25,26,27,28,29,30,31,32,33,34,35], the pancreas[36,37], the trachea[38], the kidney[39], the testis[40,41], the uterus[42,43,44], the adrenal gland[45,46], the brain[47], and the eyes of chickens[48,49,50,51] and mice[52,53]. The relationship between the intramitochondrial DNA and RNA syntheses and cell cycle was formerly studied and it was clarified that the intramitochondrial DNA and RNA syntheses were performed without any nuclear involvement[8].

On the other hand, the protein synthesis in various cells has also been extensively studied using both biochemical and morphological procedures[1,2,3,4,19,20,21,36,44,54,55,56,57,58,59,60,61,62,63]. However, the relationship between the protein synthesis in mitochondria and the aging of individual animals has not yet been clarified. This paper deals with the relationship between the protein synthesis in hepatocyte mitochondria and aging of mice *in vivo* at various ages by means of EM radioautography as a part of serial studies on special cytochemistry[64] and radioautographology[65].

### MATERIALS AND METHODS

#### The Animals

The liver tissues were obtained from respective animals in 10 small groups of normal ddY strain mice, bred and grown in our laboratory, each consisting of 3 littermates of both sexes (total 30), aged from embryonic day 19 to postnatal days 1, 3, 9, and 14, and months 1, 2, 6, 12, and 24. The embryonic age was based on observation of the vaginal plug of the female mice (vaginal plug = day 0). All the animals were housed under conventional conditions and bred with normal diet (mouse chow Clea EC2, Clea Co., Tokyo, Japan) with access to water *ad libitum*. The mean life span of the experimental animals was around 20 months on average. Thus, we used the term “senescent stage” for the animals over 12–24 months (2 years). All the procedures used in this study concerning the animal experiments were in accordance with the guidelines of the Animal Research Committee of Shinshu University School of Medicine, as well as the principles of laboratory animal care (NIH publication No. 86-23, revised 1985).

#### Radioautography

Respective animals were injected intraperitoneally with an RI-labeled amino acid, $^{3}$H-4,5-leucine (Amersham, England, specific activity 1002 GBq/m*M*), purchased from Daiichi Chemical Co., Ltd., Tokyo, Japan, 1 h before sacrifice. The dosage of injections was 370 KBq/gm body weight. In the case of embryos, $^{3}$H-4,5-leucine was injected intraperitoneally to the pregnant mother and the embryos were taken out from the uterus after the mother was sacrificed. All the animals were perfused via the left ventricles of the hearts with 0.1 *M* cacodylate-buffered 2.5% glutaraldehyde under Nembutal (Abbott Laboratories, Chicago, IL) anesthesia. The right medial lobes of the livers were excised and three small pieces of the liver tissues (1 mm × 1 mm × 1 mm) were immersed in the same fixative at 4˚C for 1 h, followed by postfixation in 1% osmium tetroxide in the same buffer at 4˚C for 1 h, dehydrated in graded series of ethanol and acetone, and embedded in epoxy resin (Epok 812, Oken, Tokyo, Japan).

Thick sections (1-µm thickness) from respective specimens were cut in sequence on a Porter-Blum MT-2B ultramicrotome (Dupont-Sorvall, Newtown, MA) using glass knives, collected on glass slides, coated with Konica NR-M2 radioautographic emulsion (Konica, Tokyo, Japan) by a dipping method[23,36,65] for light microscopy, exposed for 4 weeks at 4˚C, developed in SDX-1 developer at 20˚C for 5 min, fixed in acid fixer, stained in 0.1% toluidine blue/0.1*M* sodium solution. On the other hand, semithin sections (0.2-µm thickness) were cut in sequence from the intermediate zone of respective hepatic lobules on the same ultramicrotome, collected on collodion-coated copper grid meshes (VECO, Eerbeek, Netherlands) in order to shorten the exposure time for EM radioautography, coated with Konica NR-H2 radioautographic emulsion (Konica, Tokyo, Japan) by a wire-loop method[22,23,36,65], exposed for 10 months at 4˚C, developed in phenidon developer at 16˚C for 1 min after 30-sec gold-latensification in freshly prepared gold thiocyanate solution, and stained with lead citrate solution for 3 min. The light microscopic radioautograms were examined and photographed with an Olympus Vanox AHB-LB light microscope (Olympus, Tokyo, Japan), while the EM radioautograms were examined in either a Hitachi H-700 electron microscope (Hitachi, Tokyo, Japan) at an accelerating voltage of 200 kV or a JEOL JEM-4000EX electron microscope (JEOL, Tokyo, Japan) at an accelerating voltage of 400 kV for observing thick specimens.

### Quantitative Analysis of Electron Micrographs

Twenty electron radioautograms showing cross-sections of mononucleate hepatocytes from each group, based on the EM photographs taken at random after observation on 100 hepatocytes at least from respective animals, were analyzed to calculate the number of silver grains over mitochondria by visual grain counting. The number of mitochondria per cell profile area as well as the number of mitochondria labeled with silver grains showing $^{3}$H-leucine incorporation were calculated, respectively. In order to estimate the background fog, the number of silver grains in respective radioautograms in 10 circles with the same area size as a mitochondrion selected at random on the plastic sections outside of cell body coated with radioautographic emulsions were counted. The average number of silver grains per mitochondrial area was 0.01–0.03/area in respective groups. Then, we considered that almost no chemographic effects of hepatocyte sections as well as radioautographic procedures were detected. Therefore, the grain count in each specimen was not corrected. Thus, the mitochondrion that was labeled with more than one silver grain was defined as labeled. From all the data thus obtained, the averages and standard deviations in respective aging groups were computed with a personal computer (Macintosh type 8100/100, Apple Computer, Tokyo, Japan). The data were stochastically analyzed using variance and Student's t-test. The differences were considered to be significant at $p$ value <0.01 (n = 20).

## RESULTS

### Morphological Observations

By light microscopic observation, the liver tissues from normal ddY strain mice at various ages consisted of hepatocytes, sinusoidal endothelial cells, Kupffer cells, Ito cells, bile ductal epithelial cells, and fibroblasts. The liver tissues were composed mainly of hepatocytes and various hematopoietic cells, while no lobular orientation of hepatocytes was observed at embryonic day 19 and postnatal day 1. At postnatal day 3, orientation of hepatic cells started to form hepatic lobules and the number of hematopoietic cells decreased. At postnatal day 9, hepatic lobules were completely formed and only a few of hematopoietic cells remained. From postnatal day 14 to months 1, 2, 6, 12, and 24, hepatocytes and other cell types, such as sinusoidal endothelial cells, Kupffer cells, Ito cells, bile ductal epithelial cells, and fibroblasts, appeared in the same arrangements as completed hepatic lobules. Observing the ultrastructure of hepatocytes by EM, cell organelles were not well developed at perinatal and early postnatal stages from embryonic day 19 (Fig. 1A) to postnatal days 1 (Fig. 1B), 3 (Fig. 1C), 9 (Fig. 1D), and 14 (Figs. 1E, 1F). The numbers and sizes of Golgi apparatuses, endoplasmic reticulums, and mitochondria were less and smaller at these stages as was reported previously[29,30,31,32]. However, these cell organelles appeared well developed at adult and senescent stages from postnatal months 1 (Fig. 2A) to 2 (Figs. 2B, 2C), 6 (Figs. 2D, 2E), 12 (Fig. 2F), and 24 (Fig. 2G). The numbers and sizes of these cell organelles were more and larger at these stages[29,30,31,32].

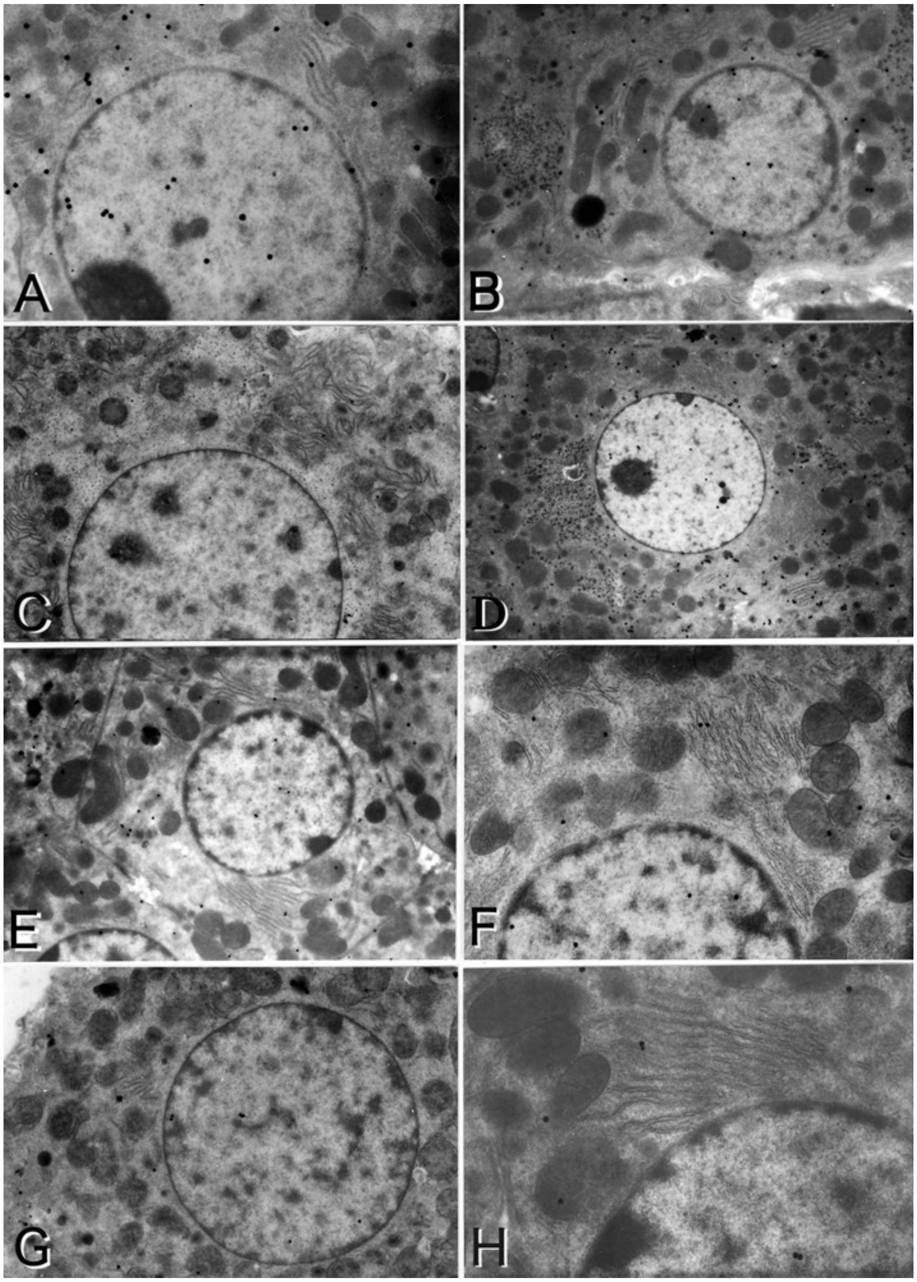

**FIGURE 1.** EM radioautograms of mononucleate hepatocytes of prenatal and postnatal newborn, juvenile, and young mice, labeled with $^{3}$H-leucine showing protein synthesis. (A) Prenatal mouse aged at embryonic day 19; several silver grains due to $^{3}$H-leucine can be seen in the nucleus, especially over the euchromatin as well as over some mitochondria and endoplasmic reticulum in the cytoplasm; original magnification ×10,000. (B) Newborn mouse aged at postnatal day 1; original magnification ×5,000. (C) Suckling mouse aged at postnatal day 3; original magnification ×6,000. (D) Weanling mouse aged at postnatal day 9; original magnification ×3,000. (E) Juvenile mouse aged at postnatal day 14; note that two silver grains are observable over the mitochondrial membranes of the two mitochondria at right as well as a silver grain over the crista of the mitochondrion at upper left; original magnification ×10,000. (F) Juvenile mouse aged at postnatal day 14; an endothelial cell can be seen at left; original magnification ×3,000. (G) Adult mouse aged at postnatal month 1; original magnification ×6,000. (H) High-power magnification of a juvenile mouse aged at postnatal day 14; a few silver grains in a few mitochondria and endoplasmic reticulum are observable; note that a silver grain in the mitochondrion at left is localized over the mitochondrial matrix; original magnification ×15,000.

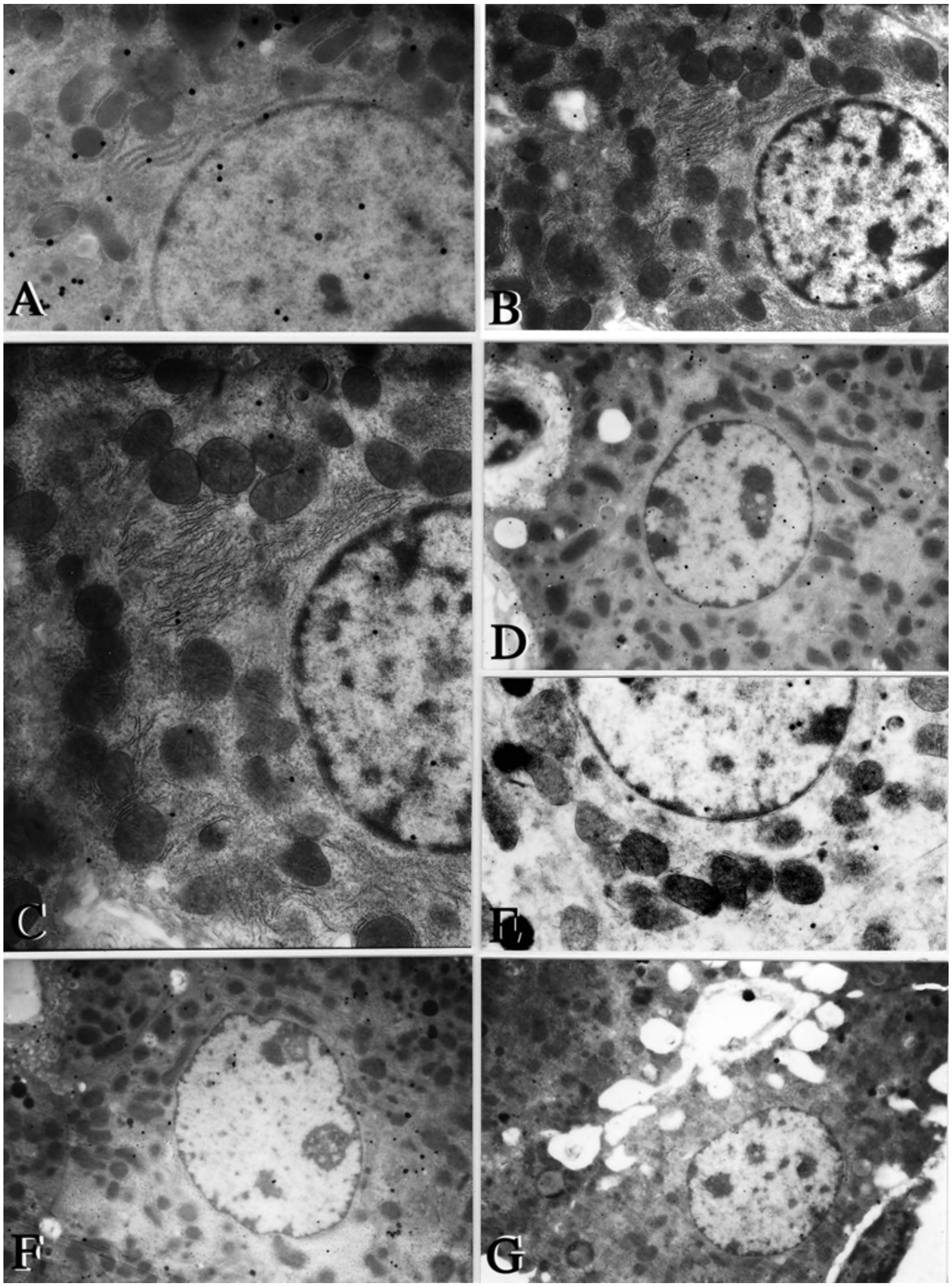

**FIGURE 2**. EM radioautograms of mononucleate hepatocytes of adult mice aged at postnatal month 1 to 24, labeled with $^{3}$H-leucine showing protein synthesis. (A) Adult mouse aged at postnatal month 1; several silver grains due to $^{3}$H-leucine can be seen in the nucleus especially over the euchromatin as well as over some mitochondria and endoplasmic reticulum in the cytoplasm; original magnification ×10,000. (B) Adult mouse aged at postnatal month 2; original magnification ×5,000. (C) High-power magnification of an adult mouse aged at postnatal month 2; silver grains can be seen localizing over mitochondrial membranes of the two mitochondria at top as well as another mitochondrion at bottom left; original magnification ×20,000. (D) Senile mouse aged at postnatal month 6; original magnification ×3,000. (E) Senile mouse aged at postnatal month 6; original magnification ×5,000. (F) Senescent mouse aged at postnatal month 12 (1 year); less silver grains can be seen as compared with the younger animals; original magnification ×3,000. (G) Senescent mouse aged at postnatal month 24 (2 years); less silver grains can be seen as compared with the younger animals; original magnification ×3,000.

### Radioautographic Observations

By observing light microscopic radioautograms, the silver grains were found over both the karyoplasm and cytoplasm of almost all the cells, not only at the perinatal stages from embryo day 19 to postnatal days 1, 3, 9, and 14, but also at the adult and senescent stages from postnatal months 1 to 2, 6, 12, and 24.

By EM observation, silver grains in the hepatocytes were observed in most hepatocytes at respective aging groups, localizing not only over euchromatin and nucleoli in the nuclei, but also over many cell organelles, such as endoplasmic reticulum, ribosomes, Golgi apparatus, and mitochondria as well as cytoplasmic matrices of mononucleate hepatocytes from perinatal stage at embryonic day 19 (Fig. 1A), postnatal days 1 (Fig. 1B), 3 (Fig. 1C), 9 (Fig. 1D), and 14 (Figs. 1E, 1F), to adult and senescent stages at postnatal months 1 (Figs. 1G, 2A), 2 (Figs. 2B, 2C), 6 (Figs. 2D, 2E), 12 (Fig. 2F), and 24 (Fig. 2G). Some binucleate hepatocytes containing two nuclei of equal size (showing that their two nuclei were sectioned at their centers) or two nuclei of unequal size, a large and a small (showing that their nuclei were not sectioned at their centers) were seldom found, which also showed silver grains localizing over euchromatin and nucleoli in the nuclei as well as many cell organelles such as endoplasmic reticulum, ribosomes, Golgi apparatus, and mitochondria. Since the number of binucleate hepatocytes found in respective aging groups was few, further quantitative analysis on the binucleate hepatocytes was omitted. As for the intramitochondrial localization of silver grains in mononucleate hepatocytes, the grains were observed to localize on the mitochondrial membranes (Figs. 1A, 1B, 1D, 1E, 1F, 2A, 2B, 2C, 2D), cristae (Figs. 1F, 1H, 2C, 2E), and matrix (Figs. 1B, 1E, 2D).

### Quantitative Analysis

#### *Number of Mitochondria per Cell*

From the results obtained, it was found that almost all the hepatocytes were labeled with silver grains showing protein synthesis in their nuclei, cytoplasm, and mitochondria. Preliminary quantitative analysis on the number of mitochondria in 20 mononucleate hepatocytes whose nuclei were intensely labeled with many silver grains (more than 10 per nucleus) and other 20 mononucleate hepatocytes whose nuclei were not so intensely labeled (number of silver grains less than 9) revealed in each aging group that there was no significant difference between the number of mitochondria, number of labeled mitochondria, and the labeling indices in both types of hepatocytes ($p < 0.01$). Thus, the numbers of mitochondria, the numbers of labeled mitochondria, and the labeling indices were calculated in 20 hepatocytes selected at random in each animal in respective aging stages regardless whether their nuclei were very intensely labeled or not. The results obtained from the total numbers of mitochondria in mononucleate hepatocytes showed an increase from the prenatal day (34.5/cell) to postnatal days 1 (44.6/cell), 3 (45.8/cell), 9 (43.6/cell), and 14 (48.5/cell), to postnatal months 1 (51.5/cell) and 2 (52.3/cell), reaching the maximum at month 6 (60.7/cell), then decreased to years 1 (54.2/cell) and 2 (51.2/cell) as shown in Fig. 3. The increase and decrease were stochastically significant ($p < 0.01$). On the other hand, binucleate hepatocytes were not analyzed because the appearances of binucleate hepatocytes were not so many in perinatal and adult stages, and enough numbers of binucleate cells were not available in respective animals in each aging group in this experiment.

#### *Mitochondrial Protein Synthesis*

The results obtained from visual counting on the numbers of mitochondria labeled with silver grains from 20 mononucleate hepatocytes of each animal labeled with $^{3}$H-leucine, demonstrating protein synthesis in 10 aging groups at perinatal stages, prenatal embryo day 19, postnatal days 1, 3, 9, and 14, months 1, 2, and 6,

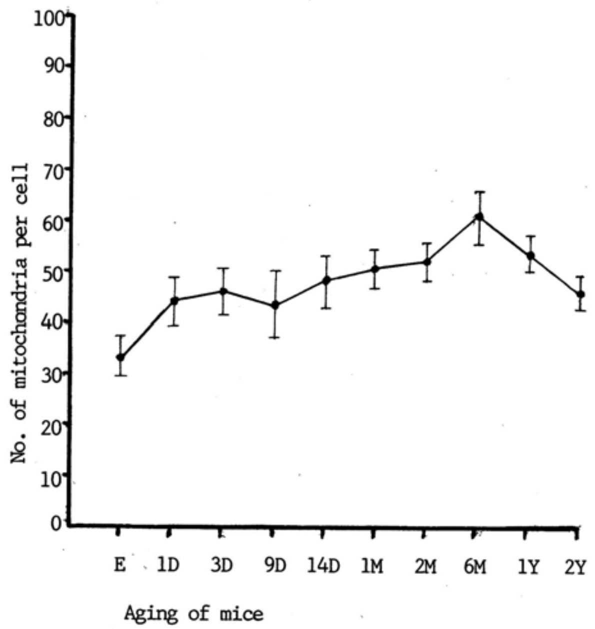

**FIGURE 3.** Histogram demonstrating aging change of the total number of mitochondria per cell profile area in mononucleate hepatocytes labeled with $^{3}$H-leucine at respective aging groups from embryonic day 19 to postnatal month 24 (mean ± standard deviation, $p < 0.01$, n = 20).

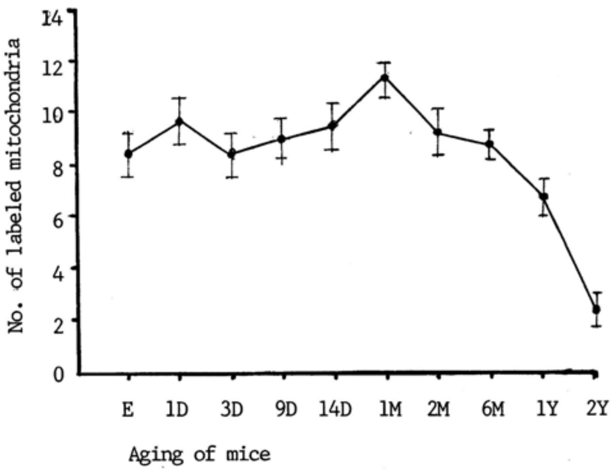

**FIGURE 4**. Histogram demonstrating aging change of the total number of mitochondria labeled with $^{3}$H-leucine showing protein synthesis per cell profile area in mononucleate hepatocytes at respective aging groups from embryonic day 19 to postnatal month 24 (mean ± standard deviation, $p < 0.01$, n = 20).

and years 1 and 2, are plotted in Fig. 4. The labeling indices in respective aging stages were calculated from the numbers of labeled mitochondria and the numbers of total mitochondria per cell which were plotted in Fig. 5. The results showed that the numbers of labeled mitochondria with $^{3}$H-leucine showing protein synthesis increased from prenatal embryo day 19 (8.3/cell) to postnatal days 1 (9.6/cell), 3 (8.1/cell), 9 (8.9/cell), and 14 (9.5/cell), and month 1 (11.2/cell), reaching the maximum, and then decreased to months

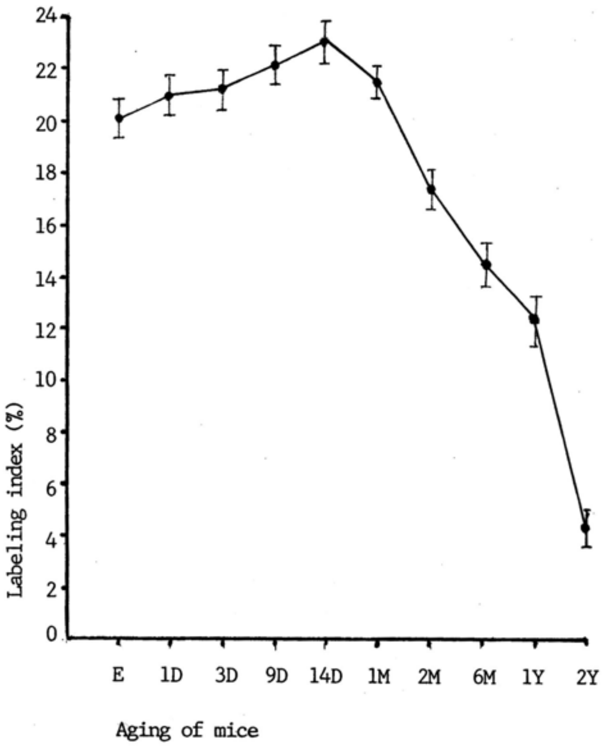

**FIGURE 5.** Histogram demonstrating aging change of the labeling index of mitochondria in mononucleate hepatocytes labeled with $^3$H-leucine showing protein synthesis (number of labeled mitochondria/number of total mitochondria) at respective aging groups from embryonic day 19 to postnatal month 24 (mean ± standard deviation, $p < 0.01$, n = 20).

2 (9.1/cell) and 6 (8.8/cell), to years 1 (6.7/cell) and 2 (2.2/cell), while the labeling indices increased from prenatal day 19 (20.1%) to postnatal days 1 (21.2%), 3 (21.6%), 9 (22.2%), and 14 (23.1%), reaching the maximum, then decreased to months 1 (21.7%), 2 (17.4%), and 6 (14.6%), and years 1 (12.4%) and 2 (4.4%). Stochastic analysis revealed that the increases and decreases of the numbers of labeled mitochondria as well as the labeling indices from the perinatal stage to the adult and senescent stages were significant ($p < 0.01$).

### DISCUSSION

Concerning the macromolecular synthesis in various cells in various organs of experimental animals observed by light and electron microscopic radioautography, it is well known that the silver grains due to the radiolabeled precursor, such as $^3$H-thymidine, $^3$H-uridine, and $^3$H-leucine, demonstrate DNA, RNA, and protein syntheses, respectively[5,6,22,23,36,65]. The present results obtained from the livers of aging mice revealed that the incorporation of $^3$H-leucine indicating protein synthesis resulted in silver grain localization over the nuclei and cell bodies of almost all mononucleate hepatocytes from perinatal animals at embryonic day 19, postnatal days 1, 2, 9, and 14, to adult and senescent stages at postnatal months 1, 2, 6, 12, and 24, which showed the localization of newly synthesized proteins in the nuclei and the cytoplasmic cell organelles including mitochondria. From the results obtained at present, the numbers of mitochondria in respective hepatocytes showed increases and decreases reaching the maxima at postnatal month 6, while the numbers of labeled mitochondria with silver grains due to $^3$H-leucine incorporation, demonstrating intramitochondrial protein synthesis, also showed increases and decreases reaching the maximum at postnatal month 1, and the labeling indices of mitochondria labeled with $^3$H-leucine showed increases and decreases reaching the maximum at postnatal day 14.

With regards to protein synthesis in mitochondria in animal cells or plastids in plant cells, many studies have recently reported various cells of some plants[66,67,68] or several kinds of animals, such as hamster[69], catfish[70], rat[71], or human patients[72,73,74] by various approaches. Most of these authors observed mitochondrial protein synthesis in various cells by means of biochemical procedures. They supposed that the proteins detected in the mitochondria of these cells were synthesized in cytoplasmic cell organelles, such as endoplasmic reticulum, and moved into mitochondria, especially mitochondrial matrices. However, no evidence showing protein synthesis in mitochondria or plastids of any kind of cells, either plants or animals, has been demonstrated *in situ* by morphological methods except several papers published from our laboratory[5,32,33,36,54,56,60,62,63]. Among these studies, we first demonstrated that the numbers of mitochondria and the numbers of labeled mitochondria incorporating $^3$H-leucine in mouse hepatocytes increased from prenatal stage to postnatal adult stage at month 1, but the labeling indices did not show any significant increase[62]. In the present study, it was clearly demonstrated that the numbers of mitochondria, the numbers of labeled mitochondria with $^3$H-leucine, and the labeling indices of mouse hepatocytes showed significant increases and decreases from perinatal stage to adult and senescent stages. The present study, showing the silver grain localization in the mitochondria of mouse hepatocytes incorporating $^3$H-leucine as observed by EM radioautography, should be the first report to demonstrate the protein synthesis in the mitochondria of animal cells *in situ* in connection to animal aging from perinatal stages to postnatal adult and senescent stages as studied systematically. It should be worthy of notice that the silver grains localized over the mitochondrial membranes, cristae, and matrices of all hepatocytes at various ages. The results indicated that the protein synthesis as shown by $^3$H-leucine incorporations demonstrated the synthesis of structural proteins in the mitochondrial membranes, cristae, and matrices.

However, it is also worthy of notice that the silver grains demonstrated in mitochondria contained both the newly synthesized proteins in mitochondria themselves and the newly synthesized proteins outside mitochondria, such as endoplasmic reticulum. In order to differentiate the two kinds of proteins, the newly synthesized proteins in mitochondria themselves and the newly synthesized cytoplasmic proteins in endoplasmic reticulum which were transported into mitochondria, it should be necessary to carry out another experiment by demonstrating protein synthesis using different specific inhibitors such as inhibitors of mitochondrial proteins, or inhibitors of mitochondrial protein transporters, or inhibitors of cytoplasmic protein synthesis. It requires further investigations in the future.

On the other hand, the problems concerning the heterogeneity of hepatocyte localization in hepatic lobules should also be considered. It is well known that functional differences exist among the hepatocytes in the hepatic lobules, from central to intermediate and peripheral zones, as well as different ploidies from diploid to tetraploid and even octaploid nuclei[25,29,30,31,32,33,34,64,65].To avoid these differences, the present experiment was undertaken to observe only the hepatocytes in the intermediate zone of the hepatic lobules so that the periportal and perivenous effects were excluded. With regard to the ploidy problems, it is theoretically impossible to differentiate the hepatocytes into diploid, tetraploid, or even to octaploid nuclei on EM radioautograms after the hepatocytes were sectioned and processed for radioautography. In order to determine the ploidy of hepatocytes, the hepatocytes with intact nuclei without sectioning should be observed by microspectrophotometry before radioautography[64]. Thus, the present experiment was carried out without microspectrophotometry. However, it is believed that most hepatocytes observed in the intermediate zone of the hepatic lobules were mainly diploid, while polyploidy nuclei such as tetraploid and octaploid were less than 20%, if any[64]. Therefore, it is believed that the results obtained at present represent the average data from diploid hepatocytes.

As for the macromolecular synthesis in various cells in various organs of experimental animals observed by EM radioautography, it is well known that the silver grains due to radiolabeled $^3$H-thymidine demonstrate DNA synthesis[1,2,3,4,5,6,7,8,10,12,13,17,18,19,21,22,23,24,25,26,27,28,33,35,37,59,64,65], while $^3$H-uridine demonstrates RNA synthesis[1,2,3,4,7,8,9,10,11,12,13,14,15,16,21,22,34,36,64,65], and $^3$H-leucine demonstrates protein synthesis[5,6,7,8,9,11,12,14,15,18,19,22,23,36,56,61,65]. The previous studies obtained from the livers of aging mice revealed that silver grains incorporating either $^{3}$H-thymidine or $^{3}$H-uridine were observed not only over the nuclei of some hepatocytes, but also over the mitochondria, showing intramitochondrial DNA and RNA synthesis[33,34,35,59].

With regard to the incorporation of $^{3}$H-thymidine or $^{3}$H-uridine into mitochondria demonstrating DNA or RNA syntheses, many authors previously reported that DNA synthesis was observed by means of EM radioautography in lower organisms, such as slime mold[75,76] and tetrahymena[77], or chicken fibroblasts in tissue culture under abnormal conditions[78,79], or liver and kidney cells of chicken and mouse under normal conditions[5] as was formerly reviewed[80,81]. Likewise, RNA synthesis in *Agaricus*[82], *Ochromonas*[83], chicken liver[84,85], mouse liver[86], rat adrenal cortex[87], and human cells[7,8,9,10,11,12,13] were also demonstrated. Most of these authors, however, used old-fashioned developers consisting of methol and hydroquinone (MQ-developer), which produced coarse, spiral, silver grains, resulting in inaccurate localization over cell organelles, especially mitochondria, when observed by EM. Thus, most of these authors, except Nagata[7,8,9,10,11,12,13], showed photographs of electron radioautograms with large, spiral-formed, silver grains (2–3 μm in diameter), localizing not only over the mitochondria, but also outside the mitochondria. In order to obtain smaller, silver grains, we first used elon-ascorbic acid developer after gold latensification[7,8,9,10,11,12,13], which produced comma-shaped, smaller, silver grains (0.3–0.8 μm in diameter) much better for localization over mitochondria than spiral, silver grains (2–3 μm in diameter). Later, we used phenidon developer after gold latensification, producing dot-like, smaller, silver grains (0.2–0.3 μm in diameter), localizing only inside the mitochondria showing ultrahigh resolution of radioautograms[4,22,23,34,35,36,61,65] as shown in Figs. 1 and 2 in this study. Thus, these papers from our laboratory were the first to demonstrate intramitochondrial DNA, RNA, and protein syntheses incorporating $^{3}$H-thymidine, $^{3}$H-uridine, and $^{3}$H-leucine, respectively, with accurate intramitochondrial localization in avian and mammalian cells in 1960s and 1970s[5,6,7,8,9,10,11]. Concerning to the resolution of EM radioautography, on the other hand, several authors discussed the sizes of silver grains under various experimental conditions and calculated various values of resolutions[8,9,10,88,8990]. Those authors who used the M-Q developers maintained the resolution to be 100–160 nm[88-90], while those authors who used the elon-ascorbic acid developer[8,9,10,91] calculated it to be 25–50 nm. When we used phenidon developer at 16˚C for 1 min after gold latensification, we could produce very fine, dot-shaped, silver grains and obtained the resolution around 25 nm[21,22,23,36,92]. For the purpose of analyzing electron radioautographs, Salpeter et al.[89] suggested using the half distance and proposed using very complicated calculations through which respective, coarse, spiral-shaped, silver grains were judged to be attributable to the radioactive source in a certain territory within a resolution boundary circle. However, since we used phenidon developer after gold latensification to produce very fine, dot-shaped, silver grains, we judged only the silver grains that were located in the mitochondria that were dot-shaped and very fine to be attributable to the mitochondria without any problem with the resolution around 25 nm as was formerly discussed[8,9,10]. As for the section thickness, we used thicker semithin sections (0.2-μm thickness), which did not affect the HD value of this experiment since we used tritium as RI, which emitted beta rays with very low energy having very short range as 0.2 μm in the emulsions. Thus, the numbers of labeled mitochondria as well as the labeling indices that were calculated from the results obtained from the numbers of mitochondria over which the silver grains really existed without adding any hypothetical silver grains should be less than 10% (only several %) if added under the experimental conditions that we carried out in this experiment.

On the other hand, the incorporation of $^{3}$H-thymidine into mitochondria demonstrating DNA synthesis was formerly observed by means of EM radioautography not only in lower organisms, such as slime mold[75,76] and tetrahymena[77,93], but also in higher animals, such as chicken fibroblasts in tissue culture under abnormal conditions[78,79], or liver and kidney cells of chicken and mouse under normal conditions[5,6,94]. Then we also demonstrated intramitochondrial DNA synthesis incorporating $^{3}$H-thymidine or RNA synthesis incorporating $^{3}$H-uridine in some other established cell lines originated from human beings, such as HeLa cells[7,8,9,10] or mitochondrial fractions prepared from *in vivo* mammalian cells such as rat and mouse[2,3,4,5,6,7,8,9,10,11,12,13,14,15]. It was later commonly found in various cells and tissues *in vitro* obtained from various organs, such as the cultured human uterus cancer cells HeLa[16,19], cultured rat sarcoma cells[18], mouse liver, and pancreas cells *in vitro*[12,17], but also *in vivo* cells obtained from various organs, such as the salivary gland[24], the liver[25,26,27,28,29,30,31,32,33,34,35], the pancreas[36,37], the trachea[38], the kidney[39], the testis[40,41], the uterus[42,43,44], the adrenal gland[45,46], the brain[47], and the eyes[48,49,50,51,52,53] of chickens and mice. Thus, it is clear that all the cells in various organs of various animals synthesize DNA and RNA, not only in their nuclei, but also in their mitochondria.

The relationship between the cell cycle and the intramitochondrial DNA as well as RNA syntheses was formerly studied in synchronized cells, and it was clarified that both the intramitochondrial DNA and RNA syntheses were performed without any nuclear involvement[8]. However, the relationship between the aging of individual animals and the DNA and RNA syntheses has not yet been clarified except in a few papers recently published by Korr and associates on mouse brain[95,96,97]. They reported both nuclear DNA repair, measured as nuclear unscheduled DNA synthesis, and cytoplasmic DNA synthesis labeled with $^{3}$H-thymidine in several types of cells in brains, such as pyramidal cells, Purkinje cells, granular cells, glial cells, endothelial cells, ependymal cells, epithelial cells as observed by only light microscopic radioautography using paraffin sections. They observed silver grains over cytoplasm of these cells by light microscopy and maintained that it was reasonable to interpret this labeling as $^{3}$H-DNA outside the nuclei, which theoretically belonged to mitochondrial DNA without observing the mitochondria by EM. From the results, they concluded that distinct types of neuronal cells showed a decline of both unscheduled DNA and mitochondrial DNA syntheses with age in contrast to other cell types, glial and endothelial cells, that did not show such age-related changes without counting the number of mitochondria in respective cells or counting the number of labeled mitochondria or calculating the labeling indices of mitochondria at respective aging stages. Thus, from the statistics obtained from the cytoplasmic grain counting, their results seem not to be accurate without observing mitochondria directly by EM.

To the contrary, we first showed the relationship between the DNA synthesis and aging in hepatocytes of mice *in vivo* at various ages by means of EM radioautography, observing the small, dot-like, silver grains, due to incorporation of 3H-thymidine, which was developed with phenidon developer after gold latensification exactly localized inside the mitochondria[34]. We demonstrated that increases and decreases were observed in the mitochondrial numbers and the numbers of labeled mitochondria as well as labeling indices of DNA synthesis with $^{3}$H-thymidine incorporation by direct observation on mitochondria at EM level. Likewise, we also demonstrated that increases and decreases were observed by direct observation on mitochondria at EM level and obtained accurate mitochondrial number and labeling indices labeled with $^{3}$H-uridine[35]. Thus, this paper should be the first to show the relationship between the protein synthesis and aging in hepatocytes of mice *in vivo* at various ages from prenatal to postnatal juvenile, adult, and senescent stages by means of EM radioautography, observing the small, dot-like, silver grains, due to incorporation of $^{3}$H-leucine, which exactly localized inside the mitochondria.

On the other hand, it is well known that considerable numbers of binucleate hepatocytes appear in adult and senescent stages[33,34,35,59]. We formerly observed that the quantity of DNA and RNA synthesis as expressed by grain counting in karyoplasm and cytoplasm of both mononucleate and binucleate cells in mouse hepatocytes showed increases and decreases reaching the maxima at postnatal month 2 in case of mononucleate cells and at month 6 in case of binucleate cells karyoplasm and cytoplasm[33,34,35,59]. It was noted that the differences of grain counts between the mononucleate and binucleate cells in the same aging groups were stochastically significant. These results indicated that the amount of DNA and RNA synthesized and distributed in karyoplasm and cytoplasm of each binucleate cell was much more than each mononucleate cell in respective aging groups[33-35,59]. Considering these results, there is a possibility that any differences may exist between the mitochondrial protein synthesis of mononucleate and binucleate hepatocytes as was observed in DNA and RNA syntheses. However, since the numbers of binucleate hepatocytes observed in respective aging groups were only a few and not enough to compare between the respective aging groups at the present study, this problem should be further studied in details in the future.

With regard to the relationship between these morphological changes and cellular function of hepatocytes, it can be speculated that the increases and decreases of protein synthesis reflect the quantities and activities of enzyme proteins, such as cytochrome oxidase or ATPase existing in hepatocyte mitochondria. As was already reported by several authors by biochemical studies[98,99,100,101,102], the activity of oxidative phosphorylation of hepatocytes of mice and rats decreased during aging. Therefore, the present results obtained by EM observation are in good accordance with the biochemical and physiological data. Thus, the present results should be the morphological evidence that corroborated the biochemical and physiological data[98,99,100,101,102].

Anyway, the results obtained from the liver at present should form a part of special radioautographology[65], i.e., application of radioautography to the liver, as well as a part of special cytochemistry[64], as was recently reviewed by the present author. We expect that such special radioautographology and special cytochemistry should be further developed in all the organs in the future.

From the results obtained at present, it was concluded that almost all the hepatocytes of mice at various ages, from prenatal embryos to postnatal newborn, young, adult, and senescent animals, were labeled with silver grains showing protein synthesis with $^{3}$H-leucine in their mitochondria. Quantitative analysis on the numbers of mitochondria in mononucleate hepatocytes resulted in an increase from the prenatal day to postnatal months 1, 2, and 6, reaching the maximum at postnatal month 6, then decreased to year 2. The numbers of labeled mitochondria with $^{3}$H-leucine showing protein synthesis increased from prenatal days to postnatal days, reaching the maximum at postnatal month 1, then decreased to year 2, while the labeling indices increased from prenatal stage to postnatal days, reaching the maximum at postnatal day 14, then decreased to year 2.

### ACKNOWLEDGMENTS

This study was supported in part by a Grant-in-Aids for Scientific Research from the Ministry of Education, Science and Culture of Japan (02454564) as well as a Grant for Promotion of Characteristic Research and Education from the Japan Foundation for Promotion of Private Schools (1997, 1998 1999, 2000) given to the author (T.N.). The author is grateful to Dr. Kiyokazu Kametani, Technical Official, Research Center for Instrumental Analysis, Shinshu University, for his technical assistance during the course of this study.
